# Timing and type of alcohol consumption and the metabolic syndrome: ELSA-Brasil

**DOI:** 10.1186/1758-5996-7-S1-A123

**Published:** 2015-11-11

**Authors:** Bruna Angelo Vieira, Vivian Cristine Luft, Lloyd E Chambless, Maria Inês Schmidt, Dora Chor, Sandhi Maria Barreto, Bruce Bartholow Duncan

**Affiliations:** 1 Universidade Federal do Rio Grande do Sul, Porto Alegre, Brazil

## Background

The prevalence of the metabolic syndrome is rising worldwide. Its association with alcohol intake is controversial, and data is sparse concerning the influence of drinking during, as opposed to outside of meals.

## Objective

We aimed to investigate the associations of different aspects (quantity, predominant beverage and moment of consumption) of alcohol consumption with the metabolic syndrome and its components.

## Materials and methods

In cross-sectional analyses of 14570 individuals who participated in the ELSA-Brasil baseline, we fitted logistic regression models to investigate interactions between the quantity of alcohol, predominant beverage type (wine, beer or other), and principal moment of consumption with respect to meals in the association of alcohol consumption with the metabolic syndrome.

## Results

In analyses adjusted for sex, age, skin color/race, smoking, body mass index, educational level, per capita income and socioeconomic class, light consumption (up to 4 doses/week), predominantly of wine and with meals was inversely associated with the metabolic syndrome (OR=0.69, 95%CI 0.57 – 0.84), elevated fasting glucose (OR=0.83, CI95% 0.70 – 0.99), elevated waist circumference (OR=0.65, CI95% 0.51-0.84) and reduced HDL-cholesterol (OR=0.63 95%CI 0.50 – 0.79), compared to abstention/occasional drinking. Drinking predominantly wine, regardless of the moment of consumption, was never significantly associated with higher odds of any component of the syndrome. On the other hand, greater consumption of alcohol (>7 doses/week), predominantly as beer, when mainly consumed outside of meals was significantly associated with the metabolic syndrome (7 to 14 doses/week: OR=1.43, 95%CI 1.18 – 1.73; more than 14 doses/week: OR=1.70, 95%CI 1.35 – 2.15) and with syndrome components, except for low HDL-cholesterol.

## Conclusion

The association of alcohol consumption with the metabolic syndrome and many of its individual components differed markedly by predominant beverage and the consumption's relationship to meals. Protective associations were generally seen when consumption was with meals and in smaller quantities, and when it was wine; risk associations were generally seen with greater quantities and consumption outside of meals.

